# CD86 Is a Selective CD28 Ligand Supporting FoxP3+ Regulatory T Cell Homeostasis in the Presence of High Levels of CTLA-4

**DOI:** 10.3389/fimmu.2020.600000

**Published:** 2020-12-08

**Authors:** Neil Halliday, Cayman Williams, Alan Kennedy, Erin Waters, Anne M. Pesenacker, Blagoje Soskic, Claudia Hinze, Tie Zheng Hou, Behzad Rowshanravan, Daniel Janman, Lucy S. K. Walker, David M. Sansom

**Affiliations:** ^1^ Institute of Immunity and Transplantation, University College London, London, United Kingdom; ^2^ Institute of Liver and Digestive Health, University College London, London, United Kingdom

**Keywords:** regulatory T cells, costimulation, CD86, CD80, CTLA-4, homeostasis, CD28

## Abstract

CD80 and CD86 are expressed on antigen presenting cells and are required to engage their shared receptor, CD28, for the costimulation of CD4 T cells. It is unclear why two stimulatory ligands with overlapping roles have evolved. CD80 and CD86 also bind the regulatory molecule CTLA-4. We explored the role of CD80 and CD86 in the homeostasis and proliferation of CD4+FoxP3+ regulatory T cells (Treg), which constitutively express high levels of CTLA-4 yet are critically dependent upon CD28 signals. We observed that CD86 was the dominant ligand for Treg proliferation, survival, and maintenance of a regulatory phenotype, with higher expression of CTLA-4, ICOS, and OX40. We also explored whether CD80-CD28 interactions were specifically compromised by CTLA-4 and found that antibody blockade, clinical deficiency of CTLA-4 and CRISPR-Cas9 deletion of CTLA-4 all improved Treg survival following CD80 stimulation. Taken together, our data suggest that CD86 is the dominant costimulatory ligand for Treg homeostasis, despite its lower affinity for CD28, because CD80-CD28 interactions are selectively impaired by the high levels of CTLA-4. These data suggest a cell intrinsic role for CTLA-4 in regulating CD28 costimulation by direct competition for CD80, and indicate that that CD80 and CD86 have discrete roles in CD28 costimulation of CD4 T cells in the presence of high levels of CTLA-4.

## Introduction

Costimulatory signals, in addition to T cell receptor (TCR) engagement, are essential for T cell survival, expansion and acquisition of effector functions (1). CD28 is a major costimulatory receptor for both CD4 and CD8 T cells supporting these functions *via* the engagement of two ligands, CD80 and CD86 (2, 3). CD80 and CD86 arose from a gene duplication event during mammalian evolution (4, 5) but have undergone significant sequence divergence, retaining only 26% amino acid sequence identity (6). Despite this divergence both ligands retain binding to two receptors that possess opposing functions, the activating receptor CD28 and the regulatory receptor CTLA-4 (7). Thus, the functional differences between CD80 and CD86 are of considerable biological interest but remain largely obscure.

Some evidence suggests that CD80 and CD86 have overlapping roles, where both ligands are able to costimulate T cell proliferation, IL-2, and IFN-γ production (6, 8). In addition, deficiency of either ligand alone in mice produces a mild phenotype with modest reductions in T cell costimulation but normal CD4 T cell frequencies and immunoglobulin levels (9, 10) suggesting that they can compensate for each other. These limited functional differences have led to the general perception that CD80 and CD86 have overlapping or possibly redundant roles (8, 9).

Nonetheless, the significant sequence divergence between CD80 and CD86 argues against redundancy and differences in their biology have also been observed. For example, CD80-/- mice mount humoral and cytotoxic T cell responses to antigen or DNA vaccination, which are only modestly reduced compared to wild-type. In contrast, CD86-/- mice fail to undergo isotype class switching, form germinal centers following antigenic challenge in the absence of adjuvant and have impaired T cell proliferative and cytotoxic responses (9, 10). Additionally, T cells costimulated with CD86 deficient APCs produce lower levels of IL-2, IFN-γ, and IL-4 compared to CD80 deficient APCs (11). Furthermore, CD86 has been suggested to be the dominant costimulatory ligand, compared to CD80, for T cell allo-responses stimulated by human dendritic cells *in vitro* (12).

The expression patterns of CD80 and CD86 also differ, with CD86 often constitutively expressed on antigen presenting cells whereas CD80 availability increases following activation (13–17). There is also clear differential expression in certain cell types, with CD80 selectively expressed on some B cell subsets (18, 19) and medullary thymic epithelial cells (20), whereas CD86 is found alone on human monocytes (21). Together, these observations suggest that CD80 and CD86 functions are not identical and that perhaps CD86 may be the more important costimulatory ligand. This is unexpected given its lower affinity for CD28 which is ~10 fold lower than CD80 for CD28 (7). These affinity differences may be amplified further in cell membranes where CD86 is present as a monomer but CD80 is a non-covalent dimer (22). Indeed, the avidity of CD80 dimers for the CTLA-4 dimer is estimated to increase receptor-ligand interactions by several orders of magnitude (7).

The most obvious biological setting where the balance between CD28 and CTLA-4 binding to ligands may be relevant, is on regulatory CD4 T cells (Treg). Treg are critical regulators of the immune system (23) and have an absolute dependence upon CD28 costimulation in the thymus and periphery for their homeostasis (24–28) yet they constitutively express high levels of CTLA-4 (29, 30). Since CTLA-4 has markedly higher affinity for both CD80 and CD86, than CD28 (7) it is unclear how Treg access a CD28 signal in the presence of this high affinity competing receptor. We sought to test whether the presence of CTLA-4 on Treg might potentially discriminate the different functional capabilities of CD80 and CD86. We therefore carried out a detailed analysis of Treg responses to CD80 and CD86 ligands alone. During these experiments we observed that Treg were selectively dependent upon CD86 for their survival, proliferation and activation state. More effective CD28 signaling by CD86 was suggested by the maintenance of CTLA-4, ICOS and OX40 expression. The impaired ability of CD80 to support these functions was due to its strong bias for CTLA-4 interactions, which were mitigated in settings of Treg CTLA-4 deficiency. Accordingly, reduced CTLA-4 expression resulted in improved Treg responses *via* CD80-CD28 interactions. Taken together these data show that the relative bias of CD80 towards CTLA-4 impairs its function as a CD28 ligand on Treg, resulting in CD86 being the preferred ligand for Treg homeostasis.

## Materials and Methods

### T Cell Isolation

Blood samples from healthy subjects were obtained from fresh human leukocyte cones (NHS Blood and Transplant, UK). Mutations in CTLA-4 or LRBA were identified in the course of routine clinical investigation of patients and CTLA-4 testing carried out as part of confirmatory analysis.

Peripheral blood mononuclear cells (PBMCs) were isolated by dilution of leukocyte cones 1:6 and fresh blood 1:2 with phosphate buffered saline, layered over Ficoll-Paque PLUS (GE Healthcare) and centrifuged at 1060 g for 25 min. The PBMC layer was collected, washed three times in PBS and resuspended in 0.5% bovine serum albumin, 2 mM EDTA in PBS.

Total CD4+ T cells and memory CD4+CD45RO+ T cells were isolated by immunomagnetic negative selection from PBMCs using EasySep Human CD4+ T Cell Enrichment and memory CD4+ T Cell Enrichment Kits (Stemcell Technologies) following the manufacturer’s instructions. Treg were purified from total CD4 T cells using the EasySep Human CD4+CD127loCD49d- Regulatory T Cell Enrichment Kit (Stemcell Technologies) following the manufacturer’s instructions.

### Ethical Approval

The use of healthy donor and patient blood samples was performed under institutional ethical approval.

### T Cell Culture

T cell culture was performed in RPMI 1640 (Invitrogen) supplemented with 10% fetal calf serum (LabTech), 2 mM L-Glutamine (Sigma-Aldrich), 1% penicillin and streptomycin (Invitrogen) at 37°C, 95% humidity, 5% CO2. Assessment of memory T cell proliferation and phenotype was undertaken following incubation with 5μM Cell Trace Violet (ThermoFisher Scientific) in PBS for 20 min at 37°C, then washing 3 times in media. Where indicated, the following antibodies and cytokines were added: 2 μg/ml anti-CD80 (R&D systems), 2 μg/ml anti-CD86 (R&D systems), 20 μg/ml anti-CTLA-4 (Ticilimumab) (a gift from Pfizer), 1 μg/ml anti-CD28, recombinant human Interleukin 2 (PeproTech).

### CHO and DG75 Cell Culture

CHO cells expressing human CD80 or CD86, both C terminally tagged with Green Fluorescent Protein (GFP), or human CD32 [Fcγ Receptor II (FcR)] were cultured in Dulbecco’s modified Eagle media (DMEM) (Invitrogen) supplemented with 10% fetal calf serum (Sigma-Aldrich), 2 mM L-glutamine and 1% penicillin and streptomycin and passaged approximately every 72 h. DG75 B cells were transduced with human CD80-GFP or human CD86-GFP constructs, after deletion of endogenous CD80 and CD86 by CRISPR/Cas9, and cultured in RPMI supplemented 2 mM L-Glutamine (Sigma-Aldrich), 10% fetal calf serum (LabTech) and 1% penicillin and streptomycin (Invitrogen) at 37°C, 95% humidity, 5% CO2 and were passaged approximately every 72 h. Equivalent levels of CD80 and CD86 expression in CHO and DG75 cell lines was confirmed by comparing GFP levels by flow cytometry (**Figures S1A, B**).

### T Cell Stimulation Assays

CHO cells expressing human CD80, CD86, or human FcγRII (CD32) or CHO-blank cells were trypsinized, fixed in 1 ml 0.025% glutaraldehyde (Sigma Aldrich) for 3 min then washed four times in RPMI. 1 × 10^5^ Memory CD4 T cells or Treg were incubated with CHO-CD80, CHO-CD86, or CHO-blank cells at 1:10 ratio or 1:2.5 with CHO-FcR in round-bottomed 96-well plates for 5 days with 0.5 μg/ml anti-human-CD3 (OKT3) (BioXCell), or for CHO-FcR experiments with anti-human CD3 or anti-human-CD28 (clone 9.3) (BioXCell) antibodies at 1 μg/ml, after which proliferation and phenotype were assessed by flow cytometry.

For T cell stimulation with DG75 cells, 1 × 10^5^ purified total memory CD4 T cells were incubated 1:1 with glutaraldehyde fixed DG75 cells expressing human CD80, human CD86 or blank DG75 cells, and 1 μg/ml anti-human-CD3 in round bottom 96-well plates for 5 days prior to flow cytometry.

### Bead Stimulation of T Cells From CTLA-4 Deficient Patients

To assess CTLA-4 levels in patient samples 2.5 × 10^5^ total CD4 T cells were plated in round-bottomed 96 well plates with 2:1 T cells:human T activator anti-CD3/anti-CD28 Dynabeads (Gibco) for 16 h prior to flow cytometry [as in (31)].

### CRISPR-Cas9 Deletion of CTLA-4

CD4+CD25+ T cells were enriched from fresh human leukocyte cones by using a RosetteSep CD4+ T cell enrichment cocktail (Stemcell Technologies) and CD25 Microbeads (Miltenyi Biotech). CD4+CD25hiCD127lo Treg were then flow sorted using a FACSAria (BD Biosciences). Purified Treg were pre-activated in OpTmizer medium, supplemented with 1% penicillin and streptomycin, and 1% GlutaMAX (all Gibco) with 6.25 µl/ml CD3/CD28 tetramers (ImmunoCult; Stemcell Technologies) for 2 days with 1000 IU/ml IL-2.

sgRNAs were designed using http://crispr/mit/edu/ and synthesized using the EnGen sgRNA Synthesis Kit (NEB) and purified with the RNA Clean and Concentrator Kit (Zymo Research) according to the manufacturers’ instructions. Treg underwent electroporation using the Neon Transfection System (Invitrogen) (voltage: 2000 v, width 20 ms, 2 pulses) with 20 pmol Cas9 (Invitrogen) and 500 ng CTLA-4 specific sgRNA. Untreated control Treg did not receive sgRNA, Cas9 or undergo electroporation. Treg were expanded by co-plating with 4 × 10^4^ irradiated L cells as feeder cells, 100 ng/ml anti-CD3 (OKT3) and 1000 IU/ml IL-2 in 96-well plates. Treg were split as required and after at least 6 days Treg were rested for 24 h in fresh media with 100IU IL-2, then underwent quantification of total CTLA-4 levels by flow cytometry and used in stimulation assays, as above.

### Proliferation Analysis and Absolute Cell Number Determination

T cell survival was assessed with a fixable viability dye (eBioscience) prior to antibody staining for flow cytometry. Determination of absolute T cell numbers by flow cytometry was enabled by the addition of 1 × 10^4^ AccuCheck counting beads (Invitrogen) at the time of flow cytometry acquisition. Proliferation was assessed by CTV dilution utilizing the Proliferation Analysis function of FlowJo (Version 9.9.4, Treestar), whereby each generation was identified by the presence of a peak of CTV fluorescence. Division index = $\sum_{\text{l}}^{i}{\text{N}}_{i}/\sum_{\text{l}}^{i}\frac{{\text{N}}_{i}}{2^{i}}$ where i = generation number, N_i_ = number of events in generation i [as per (32)].

### Flow Cytometry

Flow cytometry was performed using an LSRFortessa (BD Biosciences) and analyzed using FlowJo Version 9.9.4 (Treestar). Where median fluorescence intensity (MFI) was compared between samples application settings were used to enable comparison of raw MFI data between independent experiments. For surface staining, cells were incubated with anti-CD4 AF700 (clone RPA-T4; BD Biosciences), anti-CD25 BV605 (clone 2A3; BD Biosciences), anti-OX40(CD134) PECy7 (clone ACT35; BD Biosciences), anti-ICOS(CD278) BUV395 (clone DX29; BD Biosciences), anti-PD1(CD279) BV786 (clone EH12.2HZ; BD Biosciences). For intracellular staining, cells were fixed and permeabilized using the FoxP3 staining kit (eBioscience) and stained with anti-CD154(CTLA-4) PE (clone BNI3; BD Biosciences) or anti-CTLA-4 BV786 (clone BNI3; BD Bioscience), anti-FoxP3 APC (clone 236A/E7; eBiosciences), anti-Ki67 PECy7(clone B56; BD Biosciences).

### Statistical Analysis

Statistical analysis was performed using Prism (v.6) (GraphPad Software). Paired T tests or analysis of variance testing were used as appropriate to the experimental design. P values of ≤0.05 were considered significant. Results with statistically significant one-way ANOVA underwent multiple comparison testing with Tukey’s test.

## Results

### Treg Proliferate Preferentially in Response to Costimulation by CD86

**Figure 1:**
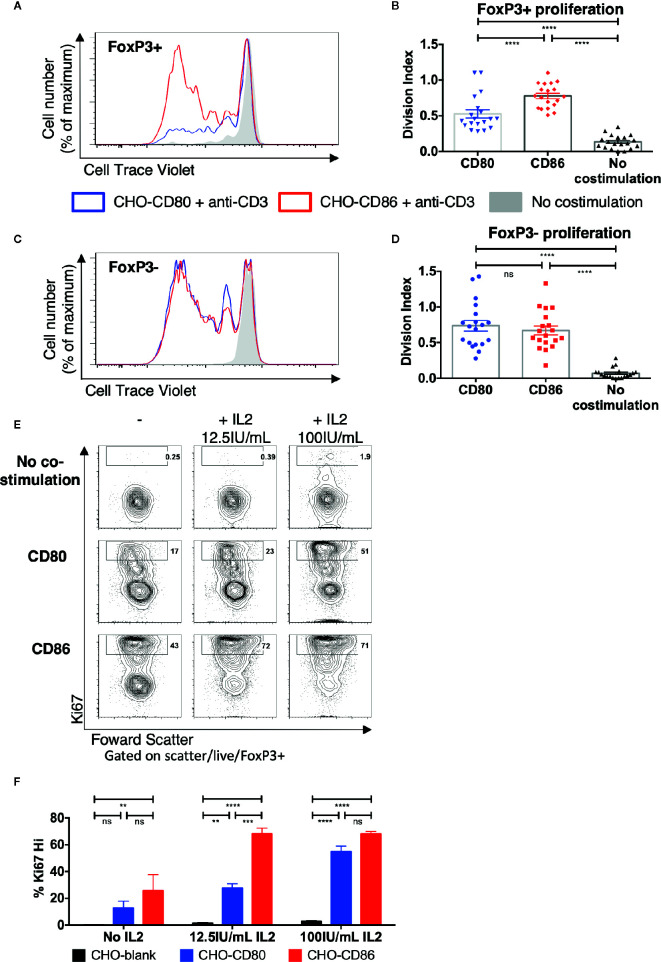
Treg selectively proliferate in response to CD86 costimulation. **(A–D)** CTV labelled total memory CD4 T cells isolated from healthy donors were cultured for 5 days with CHO-CD80, CHO-CD86, or untransduced CHO (no costimulation), at a ratio of 10 T cells to one CHO cell, with 0.5 μg/ml anti-human-CD3. **(A)** representative proliferation traces and **(B)** division indices of FoxP3+ T cells are shown. **(C)** proliferation traces and **(D)** division indices of FoxP3- conventional memory CD4 T cells (19 independent experiments). **(E**, **F)** purified Treg isolated from healthy donors were cultured with CHO-CD80, CHO-CD86, or untransduced CHO cells, at 10:1 ratio for 5 days, with 0.5 μg/ml anti-human-CD3 with increasing additional IL2. **(E)** representative Treg Ki67 expression. **(F)** percentage of Ki76+ Treg after 5 days (three independent experiments). Statistical significance of Tukey’s multiple comparisons tests: ns p>0.05, **p ≤ 0.01, ***p ≤ 0.001, ****p ≤ 0.0001 using One-way analysis of variance (ANOVA) **(B**, **D)** and two-way ANOVA **(F)**.

To investigate Treg responses following costimulation with CD80 or CD86, cell trace violet-labeled total memory CD4 T cells were stimulated with ligand-expressing transfectants in the presence of anti-CD3. After 5 days we observed clear differences where FoxP3+ cells proliferated more readily when costimulation was provided by CD86 rather than CD80 (**Figure 1A**) with the division index being significantly greater with CD86 across multiple individuals (**Figure 1B**). In contrast, when we tested the proliferation of FoxP3- conventional memory T cells (Tcon), costimulation provided by either CD80 or CD86 resulted in similar levels of proliferation across multiple donors (**Figures 1C, D**). These differences could not be accounted for by the level of expression of CD80 and CD86 as these were matched between cells (**Figure S1**). Together these observations suggested that both ligands were capable of providing comparable costimulation to conventional CD4+ T cells but that Treg were preferentially stimulated by CD86.

To explore the potential influence of IL-2 availability on this outcome and ensure that differences in response to CD80 and CD86 were in pre-existing Treg rather than preferential induction of FoxP3 expression in Tcon, Treg were purified away from conventional CD4+ cells and stimulated as before with CD80 or CD86 with or without additional IL-2. In the absence of IL-2 supplementation there was limited proliferation and both CD80 and CD86 supported low levels of Ki67 expression, albeit with a trend towards better CD86 responses. This indicated that in the mixed CD4+ cell cultures above, Treg likely utilized IL-2 produced by the conventional T cells. Accordingly, in the presence of additional IL-2 we observed a greater frequency of Ki67+ Treg costimulated by CD86 compared to CD80, particularly at lower IL-2 levels (**Figures 1E, F**). These data suggested that CD86 induced greater proliferation in purified Treg likely by enhancing the response of Treg to IL-2 required for their proliferation.

### CD86-CD28 Interactions Result in Accumulation of Greater Numbers of Treg Than CD80-CD28

**Figure 2:**
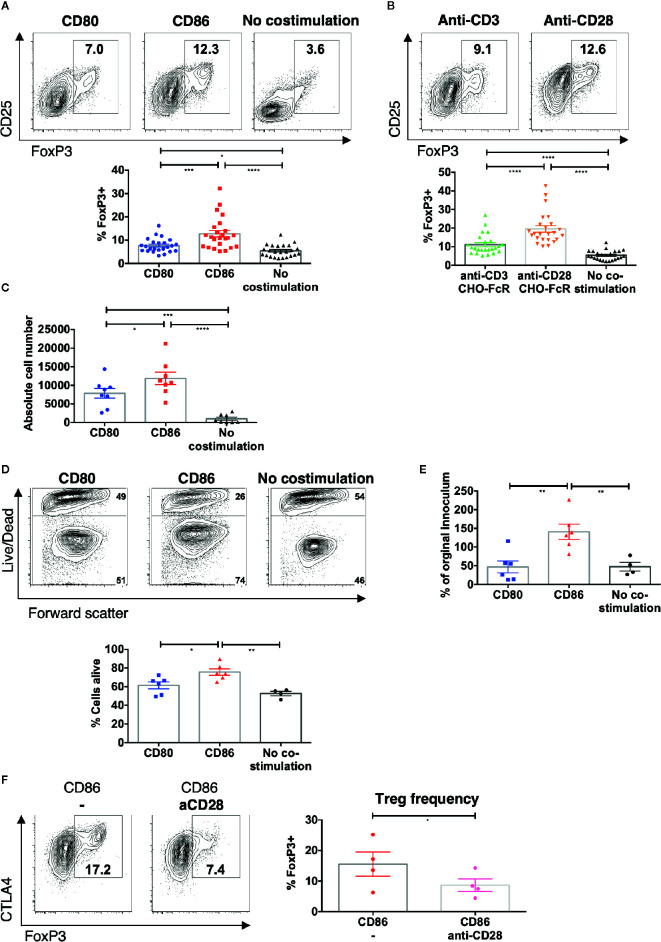
CD86-CD28 interactions drive accumulation of Treg. CTV labelled total memory CD4 T cells isolated from healthy donors were cultured for 5 days with **(A)** CHO-CD80, CHO-CD86 or untransduced CHO (no costimulation), at a ratio of 10 T cells to one CHO cell, with 0.5 μg/ml anti-human-CD3 or B) CHO-FcR at 2.5:1 ratio with 1 μg/ml anti-human-CD3 or anti-human-CD28. **(A, B)** Frequency of FoxP3+ amongst divided T cells (24 independent experiments). **(C)** absolute number of FoxP3+ cells after stimulation (under same conditions as A) (eight independent experiments). Purified Treg isolated from healthy donors were cultured with CHO-CD80, CHO-CD86 or untransduced CHO cells, at 10:1 ratio for 5 days, with 0.5 μg/ml anti-human-CD3 with 12.5 IU/ml IL2. **(D)** Representative flow cytometry plots demonstrate greater viability of Treg after 5 days with CD86. **(E)** shows the total number of Treg as a % of the starting population (six independent experiments). **(F)** CTV labeled total memory CD4 T cells isolated from healthy donors were cultured for 5 days with CHO-CD86 at 10:1 ratio with 0.5 μg/ml anti-human-CD3 and 1 μg/ml anti-human-CD28. Representative plots and graph demonstrate reduced frequency of FoxP3+ cells amongst divided T cells with blockade of CD28 (four independent experiments). One-way ANOVA with Tukey’s multiple comparisons tests **(A–D)** and paired t-test **(E)**: *p ≤ 0.05, **p ≤ 0.01, ***p ≤ 0.001, ****p ≤ 0.0001.

Given that CD86 induced greater Treg cell division we tested whether this also resulted in greater accumulation of Treg following stimulation. We therefore repeated memory CD4+ stimulation experiments and measured the percentage of Treg at day 5. CD86 stimulation resulted in a robust population of CD25hi FoxP3+ Treg among proliferating memory CD4 T cells (**Figure 2A**). Interestingly, the expansion of Treg was similar to that seen following stimulation with cross-linked anti-CD28, with higher expression of CD25 and FoxP3, in marked contrast to cross-linked anti-CD3 (**Figure 2B**), supporting the possibility that CD86 was acting as an effective ligand of CD28. The increased percentages of Treg following costimulation with CD86 interaction compared to CD80 costimulation, were also reflected in increased absolute Treg numbers following stimulation of total memory CD4 T cells (**Figure 2C**). We did not observe any significant cell death among CD4+FoxP3+ T cells stimulated with either CD80 or CD86 (**Figure S2**). Further, to confirm the origin of proliferating FoxP3+ T cells we compared CD25 depleted memory CD4 T cell populations. This revealed that following CD25 depletion, few CD25+FoxP3 Treg were identified in our cultures, indicating the above observations were due to the expansion of conventional Treg populations (**Figure S3**).

Consistent with this, stimulation of purified Treg with CD86 also gave rise to improved Treg viability (**Figure 2D**) and resulted in an expansion of Treg absolute numbers compared to CD80 (**Figure 2E**). These findings clearly indicated that CD86 supported the accumulation of Treg by enhancing their proliferation and survival.

Finally, to test whether the expansion of Treg by CD86 was a result of CD28 costimulation, we stimulated memory CD4 T cells in the presence of CD86 and a blocking anti-CD28 antibody. This revealed a clear reduction in the frequency of Treg and in Treg CTLA-4 levels indicating that Treg homeostasis was driven directly by CD86-CD28 costimulation (**Figure 2F**). Taken together these data indicate that CD86 selectively supports effective Treg proliferation and survival *via* CD28, in a manner not seen for CD80.

### CD86 Supports Treg Expression of ICOS, CTLA-4, OX40, and CD25

**Figure 3:**
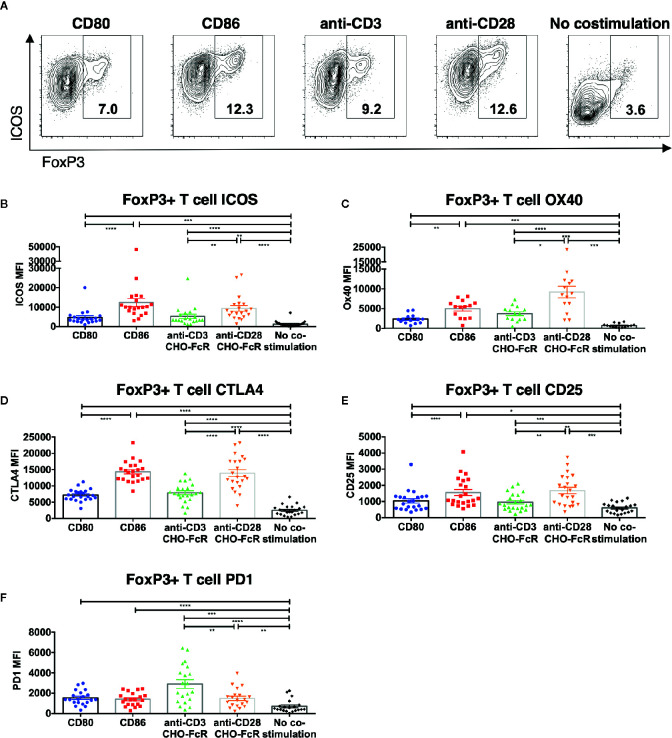
CD86-CD28 costimulation enhances expression of CD25, CTLA-4, OX40 and ICOS. CTV labelled total memory CD4 T cells isolated from healthy donors were cultured for 5 days with CHO-CD80, CHO-CD86 or untransduced CHO (no costimulation), at a ratio of 10 T cells to one CHO cell, with 0.5 μg/ml anti-human-CD3 or with CHO-FcR at 2.5:1 ratio with 1 μg/ml anti-human-CD3 or anti-human-CD28. After 5 days levels of **(A, B)** ICOS, **(C)** OX40, **(D)** CTLA4, **(E)** CD25 and **(F)** PD1 in divided CD4+FoxP3+ T cells were assessed by flow cytometry. **(A)** representative flow cytometry plots showing ICOS expression. **(B–F)** aggregate data from 22 independent experiments. One-way ANOVA with Tukey’s multiple comparisons tests: *p ≤ 0.05, **p ≤ 0.01, ***p ≤ 0.001, ****p ≤ 0.0001.

We next investigated how CD86 costimulation influenced Treg phenotype. We observed that ICOS was present on dividing Treg following CD86 costimulation at a significantly higher level compared to CD80 (**Figures 3A, B**), and was again similar to that observed with cross-linked CD28 stimulation. Similar patterns were also observed for OX40, CTLA-4 and CD25 expression in dividing Treg (**Figures 3C–E** and **Figure S4**). Notably, PD1 expression was not obviously different for either CD80 or CD86 costimulation, but was expressed most strongly in response to a strong, cross-linked TCR signal, consistent with PD1 expression being preferentially driven by TCR rather than CD28 (**Figure 3F** and **Figure S4D**). Thus, CD86 stimulation selectively supported expression of phenotypic markers in a manner similar to anti-CD28 costimulation and distinct from CD80.

**Figure 4:**
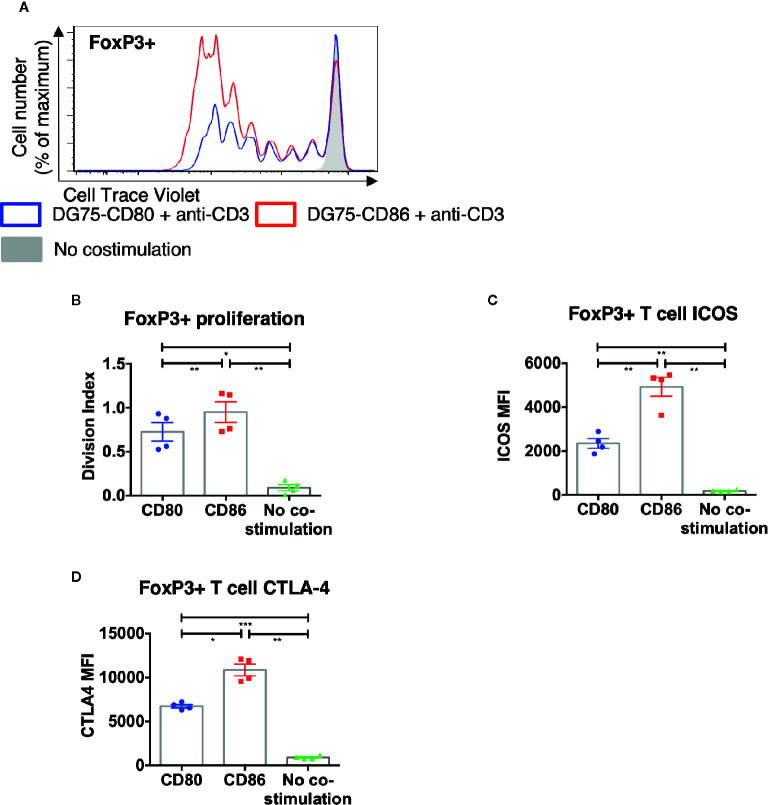
CD86-CD28 costimulation from human model APCs enhances T cell proliferation and expression of CTLA-4 and ICOS. CTV labelled total memory CD4 T cells isolated from healthy donors were cultured for 5 days with DG75-CD80, DG75-CD86, or untransduced DG75 (no costimulation), at a ratio of 1:1 T cells to DG75, with 0.5 μg/ml anti-human-CD3. After 5 days proliferation was assessed by CTV dilution. **(A)** representative proliferation traces of FoxP3+ cells following costimulation with CD86 or CD80. **(B)** FoxP3+ T cell division indices, **(C)** ICOS and **(D)** CTLA4 expression following costimulation with CD80 or CD86. Data from four independent experiments. One-way ANOVA with Tukey’s multiple comparisons tests: *p ≤ 0.05, **p ≤ 0.01, ***p ≤ 0.001.

To ensure that the above differences between CD80 and CD86 were not simply due to their expression on CHO cells, we recapitulated the key observations using a B cell line (DG75) expressing equivalent levels of CD80-GFP or CD86-GFP (**Figure S1**). In agreement with the above data, we again observed greater proliferation of FoxP3+ CD4 T cells in response to CD86 compared to CD80 (**Figures 4A, B**) and levels of both ICOS and CTLA-4 were higher following costimulation by CD86 (**Figures 4C, D**). Accordingly, we concluded the differences observed between CD80 and CD86 were fundamental features of the ligands themselves rather than the cell type expressing them.

### Treg Phenotype, but Not Proliferation Is Dependent Upon Persistent CD86 Costimulation

**Figure 5:**
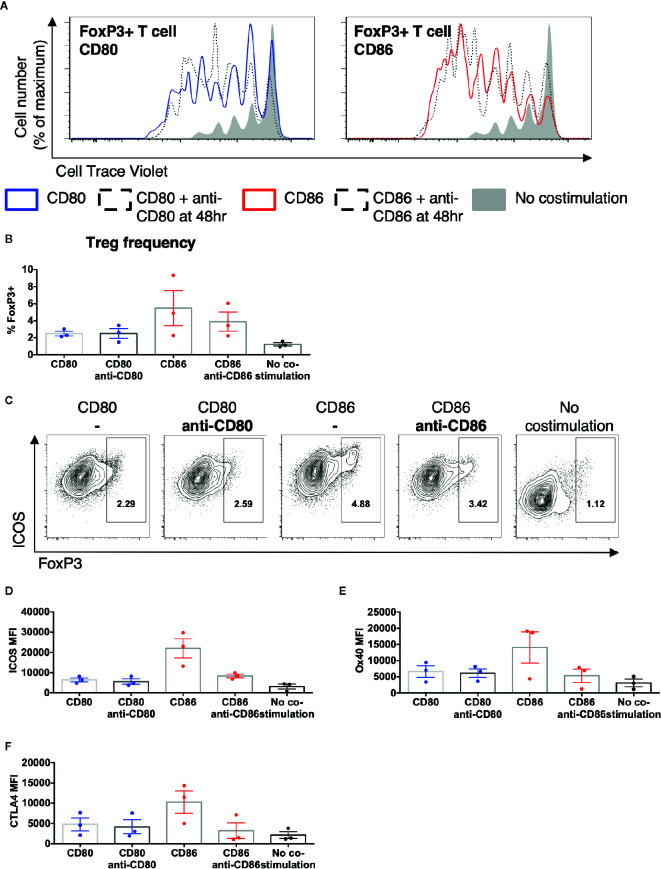
Treg phenotype is dependent upon persistent CD86 costimulation. CTV labelled total memory CD4 T cells isolated from healthy donors were cultured for 5 days with CHO-CD80, CHO-CD-86, or untransduced CHO (no costimulation), at a ratio of 10 T cells to one CHO cell, with 0.5 μg/ml anti-human-CD3 and the addition of anti-CD80 or anti-CD86 48 h. **(A)** representative proliferation traces of FoxP3+ T cells following blockade of CD80 or CD86 after 48 h of stimulation. **(B)** frequency of FoxP3+ amongst divided CD4 T cells following addition of anti-CD80 or anti-CD86 antibodies after 48 h of culture. **(C)** representative flow cytometry plots of showing ICOS expression after 5 days in divided CD4 T cells following the addition of anti-CD80 or anti-CD86 antibodies after 48 h of culture. Aggregate data for the expression of **(D)** ICOS, **(E)** OX40, **(F)** CTLA4 in divided CD4+FoxP3+ve T cells. Three independent experiments.

We next tested whether persistent CD86 or CD80 availability was required to sustain their effects on Treg. Total memory CD4 T cells were stimulated with CD80 or CD86 and after 48 h CD28 costimulation was prevented by addition of anti-CD80 or anti-CD86 antibodies. We observed that costimulation blockade from 48h had limited effect on the proliferation of Treg and Tcon (**Figure 5A**) or the frequency of FoxP3+ Treg (**Figure 5B**), since proliferative responses only require CD28 costimulation within the first 48 h (33). In contrast, following blockade of CD86 at 48 h, we observed decreased ICOS, OX40, and CTLA-4 expression in a manner not seen after blocking CD80 (**Figures 5C–F**). These data indicated that CD86 continues to provide CD28 signals to Treg beyond 48 h and that these are required for the maintenance of Treg phenotype but not their proliferation.

### CTLA-4 Prevents Treg From Receiving Costimulation From CD80, but Not CD86

**Figure 6:**
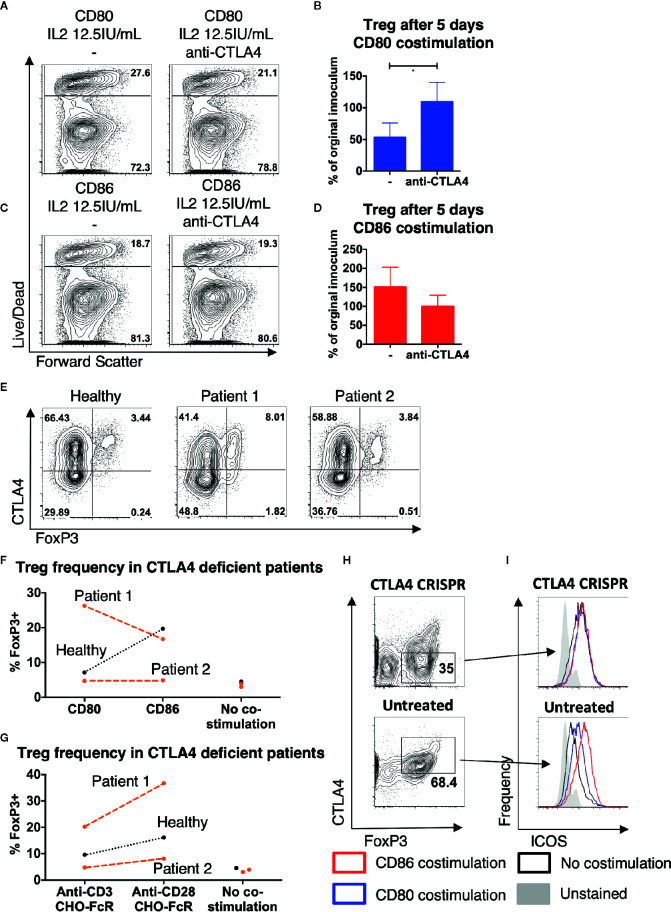
CTLA4 prevents Treg from receiving costimulation from CD80. Purified Treg isolated from healthy donors were cultured with CHO-CD80, CHO-D86, at 10:1 ratio for 5 days, with 0.5 μg/ml anti-human-CD3 and 12.5 IU/ml IL2 with anti-CTLA4 antibody. **(A, B)** cytometry plots demonstrate survival of Treg following costimulation with CD80 with- and without anti-CTLA4 antibodies and **(C, D)** similarly with CD86 costimulation. Representative plots and aggregate data from four independent experiments. **(E)** Total memory CD4 T cells were isolated from a healthy donor, a patient with LRBA deficiency (patient 1) and a patient CTLA4 deficiency (patient 2). T cells were stimulated for 16 h with 2:1 T cells: anti-CD3 and anti-CD28 coated activator beads and CTLA4 and FoxP3 expression assessed by flow cytometry, demonstrating CTLA4 deficiency in both CD4+FoxP3+ and CD4+FoxP3- T cells. **(F, G)** CTV labeled total memory CD4 T cells, isolated from patients 1 and 2 and a healthy donor, were stimulated at 10:1 ratio with CHO-CD80, CHO-CD86, or untransduced CHO (no costimulation) with 0.5 μg/ml anti-human-CD3 **(F)**, or at 2.5:1 with CHO-FcR and 1 μg/ml anti-human-CD3 or anti-human-CD28 antibodies **(G)** for 5 days. Graphs illustrate the frequency of FoxP3+ cells among the divided CD4 T cells. **(H, I)** Treg were purified from healthy donors and underwent CTLA-4 deletion by CRISPR and were then cultured 10:1 with CHO-CD80, CHO-CD86, or untransduced CHO (no costimulation) and 0.5 μg/ml anti-human-CD3 for 5 days. **(H)** demonstrates CTLA4 levels in CRISPR/Cas9 treated and untreated cells after 5 days costimulation. **(I)** Histograms demonstrating ICOS levels following either CD86 or CD80 costimulation of CTLA4-deleted or untreated, CTLA4-replete Treg (representative plot of two independent experiments). Paired t-test: *p ≤ 0.05.

Since Treg constitutively express CTLA-4, which binds to CD80 with greater affinity than to CD86, we hypothesized that the presence of CTLA-4 may be preventing CD80 mediated costimulation of Treg. We therefore tested whether blocking CTLA-4 resulted in improved Treg survival with CD80-mediated costimulation. This revealed that the survival of Treg stimulated with CD80 increased in the presence of anti-CTLA-4 antibody (**Figures 6A, B**). In contrast, anti-CTLA-4 did not enhance Treg survival when costimulation was provided with CD86 (**Figures 6C, D**). Together this suggested that CTLA-4 selectively prevented efficient Treg costimulation by CD80, which could be recovered by blockade of CTLA-4.

To further explore the role of CTLA-4 in differentiating CD80 and CD86, we hypothesized that Treg from CTLA-4 deficient patients might be more strongly costimulated by CD80 given their lower CTLA-4 expression. Interestingly, low levels of CTLA-4 due to CTLA-4 haploinsufficiency or mutations in LRBA, a putative CTLA-4 trafficking protein, have both been associated with an increased frequency of peripheral Treg (34–37). Memory CD4 T cells were therefore isolated from patient 1, who had a homozygous mutation in LRBA and patient 2, who carried a heterozygous deletion of the promotor and exon 1 of CTLA-4. Treg from both patients were observed to have reduced CTLA-4 levels (**Figure 6E**). Following 5 days of stimulation the frequency of Treg amongst dividing CD4 T cells in response to CD80 was now more similar to CD86 in the patient samples, but not in healthy controls with patient 1 showing a much better response to CD80 than CD86 (**Figure 6F**). Thus, in the setting of CTLA-4 insufficiency CD80 appeared to act as a more effective costimulator of Treg. This improvement was not due to an altered sensitivity to CD28 costimulation signals in patients as stimulation with cross-linked anti-CD28 antibody still resulted in greater Treg frequencies in both patients and controls, suggesting that Treg sensitivity to CD28 signals was similar (**Figure 6G**).

Given the difficulties in working with patient Treg in terms of disease and treatment variability, we isolated Treg from healthy subjects and deleted CTLA-4 using CRISPR. Treg were then exposed to CD80, CD86, or no costimulatory ligands for 5 days at low levels of IL-2. CTLA-4 levels were markedly reduced following CRISPR treatment (**Figure 6H**) and we gated on either CTLA-4 hi or CTLA-4 low Treg following exposure to CD80 or CD86 costimulation. Using ICOS expression as a measure of CD28 stimulation, we observed that cells expressing low CTLA-4 and costimulation from CD80 now had similar ICOS levels compared to CD86 (**Figure 6I**). However, this was not the case in non-targeted cells gated on normal levels of CTLA-4, where CD86 costimulation was more effective as expected. Taken together these limited observations support a model whereby in human Treg, decreasing levels of CTLA-4 enables Treg to receive similar costimulation signals from both CD80 and CD86. When taken together with our other observations, we conclude that CD86 is therefore the dominant ligand for CD28 in settings where T cells express high levels of CTLA-4, due to its naturally weaker interactions with CTLA-4.

## Discussion

CD28 costimulation is essential for Treg development, homeostasis and proliferation and is the outcome of four competing ligand-receptor interactions: CD80-CD28, CD86-CD28, CD80-CTLA-4, and CD86-CTLA-4. Both CD80 and CD86 are competent to provide CD28 costimulation (33, 38), however it remains unclear why two ligands exist and what functional impact their differing biophysical characteristics have on the immune system. Since Treg constitutively express CTLA-4, which has greater affinity for CD80 and CD86 than CD28, we sought to determine whether Treg might be a useful cell system in which to distinguish the capabilities of the two ligands.

Our data showed that whilst CD80 and CD86 were both capable of providing robust costimulation for proliferation of conventional CD4+ T cells, Treg revealed a clear advantage in the use of CD86. In contrast CD80 poorly supported CD28 costimulation of Treg, a feature which was abrogated by reducing CTLA-4 expression. These differences in costimulation ability resulted in significantly greater Treg proliferation and survival when CD28 costimulation was provided *via* CD86 along with other impacts such as increased CD25, ICOS, CTLA-4, and OX40 expression. Together these findings demonstrate that CD86 was capable of delivering a more effective CD28 costimulatory signal despite having markedly weaker affinity for CD28 than CD80.

The dependency of Treg on stimulatory signals *via* CD28 is well established, including roles in thymic selection, proliferation and survival. Accordingly, CD28-deficiency causes a profound deficit in Treg numbers (25, 27, 39) and general ligand deficiency has a similar effect (24, 40, 41). However, what has remained unclear is whether the two CD28 ligands are equivalent in supporting Treg. Here our data clearly show that CD86 is superior to CD80 in supporting Treg homeostasis in humans.

That Treg can derive a stronger CD28 signal from CD86, when its affinity for CD28 is at least 10-fold lower than CD80 (7), is at first sight paradoxical. However, ligand binding to CD28 is counterbalanced by competitive binding to CTLA-4. This is particularly true for Treg, where both CD28 and CTLA-4 are both constitutively expressed. In contrast, conventional T cells express CD28 but not CTLA-4 during their primary encounters with ligand-bearing APCs. Thus, priming of T cell proliferation where CTLA-4 is absent fails to reveal major differences between the two ligands. However, in the presence of CTLA-4 there is an attenuating effect on CD28 signaling that is much more evident for CD80 compared to CD86. Evidence for the counterbalancing effect of CTLA-4 is seen in settings of CTLA-4 deficiency, which can lead to overt expansions of Treg cells, albeit with compromised functional capacity (34–37).

Whilst CD80 and CD86 both bind to CTLA-4 more strongly compared to CD28 it is clear that CD80 has a much stronger relative bias for CTLA-4 over CD28 (7). Conversely, CD86 is less restrained by CTLA-4 and therefore, in principle, has improved access to CD28 when both receptors are co-expressed. Our data indicate that this greater bias for CD28 results in CD86 being a more effective ligand for supporting Treg homeostasis. We also found that the relatively impaired function of CD80 could be reversed by blockade of CTLA-4, resulting in enhanced Treg survival and ICOS expression. Thus, expression of CTLA-4 on Treg prevents effective costimulation *via* CD80, but has a much more limited impact on CD86. The previous demonstration that CD80 selectively recruits CTLA-4 to the immune synapse whereas CD86 selectively recruits CD28 (42), may reflect these biophysical biases. It is important to recognize, however, that the functional differences between CD80 and CD86 are not absolute but reflective of their relative biases towards CTLA-4 and CD28, respectively.

Our results therefore appear to be a demonstration of the “cell-intrinsic” competition between CD28 and CTLA-4 that has been widely proposed, but where there is as yet little functional evidence. This contrasts with the more obvious “cell-extrinsic” functions of CTLA-4 such as those mediated by trans-endocytosis and implicated in Treg suppressive capability (43, 44). Importantly, the intrinsic vs. extrinsic effects of CTLA-4, therefore, do not relate simply to conventional compared to Treg functions, respectively. Accordingly, cell-intrinsic effects can be seen on Treg and extrinsic functions of CTLA-4 can be also observed in conventional cells (45). Here, our data support the concept that intrinsic competition between CD28 and CTLA-4 on Treg dictates the preference for CD86 as a costimulatory ligand, in line with biophysical predictions (7).

Further support for the effect of intrinsic competition between CD28 and CTLA-4 on Treg homeostasis comes from patients deficient in CTLA-4 and the use of CRISPR to delete CTLA-4 in primary human Treg. Here we observed that reduced expression of CTLA-4 made responses to CD80 and CD86 more comparable, indicating that it is the presence of CTLA-4 that prevents CD80 from providing effective CD28 costimulation. It might therefore be predicted that expanded populations of Treg that are observed in patients and animal models with CTLA-4 deficiency (34–37) are unusually CD80 dependent.

The preferential role of CD86 in costimulating Treg and its relationship to IL-2 is also interesting. We observed that the ability of CD28 ligands to provide support for Treg was influenced by IL-2 availability. Furthermore, we found that Treg expressed significantly more CD25, CTLA-4 and ICOS in response to CD86 costimulation and were more responsive to low levels of IL-2. Hence it appears that CD86 may lower the IL-2 requirement for Treg by making them more sensitive, in a manner not seen for CD80. Consistent with previous data (28) it seems that effective CD28 signaling is important in the expression of high levels of CD25 which then permits increased sensitivity of Treg to IL-2, both of which are key features of Treg biology. Interestingly, the use of CD28 costimulation and IL-2 are standard strategies for growing Treg therapeutically. Notably, using CD28 antibodies to stimulate Treg, is devoid of any counterbalancing competition by CTLA-4, resulting in clear benefits for Treg homeostasis, which were similar to CD86 stimulation in our experiments.

While we have focused here upon the effects of CD80 and CD86 on Treg proliferation and phenotype, some impact was observed on conventional CD4+FoxP3- T cells. Proliferation of Tcon was similar when stimulated with CD80 and CD86, presumably due to the low levels of CTLA-4 present prior to stimulation. However, increased ICOS, OX40 and CTLA4 expression was apparent on divided Tcon stimulated with CD86, in a similar pattern to Treg, but to a much lesser extent. This, is likely due to upregulation of CTLA4 following activation, resulting in a similar but less marked CD86 preference following Tcon stimulation. In our view this weaker effect is most likely due to the fact that levels of CTLA-4 remain substantially lower on activated Tcon compared with Treg. Nonetheless, the impact of this upon Tcon phenotype indicates that the preferential utilization of CD86 likely relates to all CTLA-4+ CD28+ cells depending on the level of CTLA-4 expression.

In summary, we have demonstrated that Treg have a selective preference for CD86 to provide costimulation *via* CD28. The high levels of CTLA-4 competing for CD80 provides a plausible model for why despite its lower affinity, CD86 is the dominant CD28 ligand in this system. Whether, the dominance of CD86 as a CD28 ligand can generalized to the maintenance of other CTLA-4+ cells such as effector T cells in ongoing immune responses remains to be fully evaluated, but it is plausible that CD86 is the more important costimulatory ligand once CTLA-4 is upregulated in all settings. Thus, whilst it is clear that CD80 and CD86 have overlapping capabilities, our data now indicate that they have also evolved the ability to carry out discrete immunological functions.

## Data Availability Statement

The raw data supporting the conclusions of this article will be made available by the authors, without undue reservation.

## Ethics Statement

The studies involving human participants were reviewed and approved by London-Hampstead Research Ethics committee NHS Health Research Authority. The patients/participants provided their written informed consent to participate in this study.

## Author Contributions

NH and DS conceived and designed the project. NH, CW, AK, EW, AP, BS, and TH carried out experimental work. NH and DS analyzed and all authors interpreted the results. NH and DS wrote the manuscript and all authors edited the manuscript. All authors contributed to the article and approved the submitted version.

## Funding

NH was a recipient of a Wellcome Trust Clinical PhD studentship award (110297/Z/15/Z). CW was funded by Arthritis Research UK, grant 21147. DJ was funded by a BBSRC iCASE studentship with UCB Pharma. AK, CH, EW, and BR were funded by the Wellcome Trust (20478/Z/16). This research was supported in part by National Institutes of Health Research (NIHR) University College London Biomedical Research Centre funding to TH and the NIHR Rare Disease Translational Research Collaboration. LSKW and DMS were supported by the MRC (MR/N001435/1). DMS is a recipient of a Wellcome Trust Investigator Award (204798/Z/16/Z). The funder bodies were not involved in the study design, collection, analysis, interpretation of data, the writing of this article or the decision to submit it for publication.

## Conflict of Interest

The authors declare that the research was conducted in the absence of any commercial or financial relationships that could be construed as a potential conflict of interest.
