# Supplementary figures and images for: CD86 Is a Selective CD28 Ligand Supporting FoxP3+ Regulatory T Cell Homeostasis in the Presence of High Levels of CTLA-4
Source: Front Immunol. 2020 Dec 8;11:600000. doi: 10.3389/fimmu.2020.600000 (PMC7753196; doi:10.3389/fimmu.2020.600000)

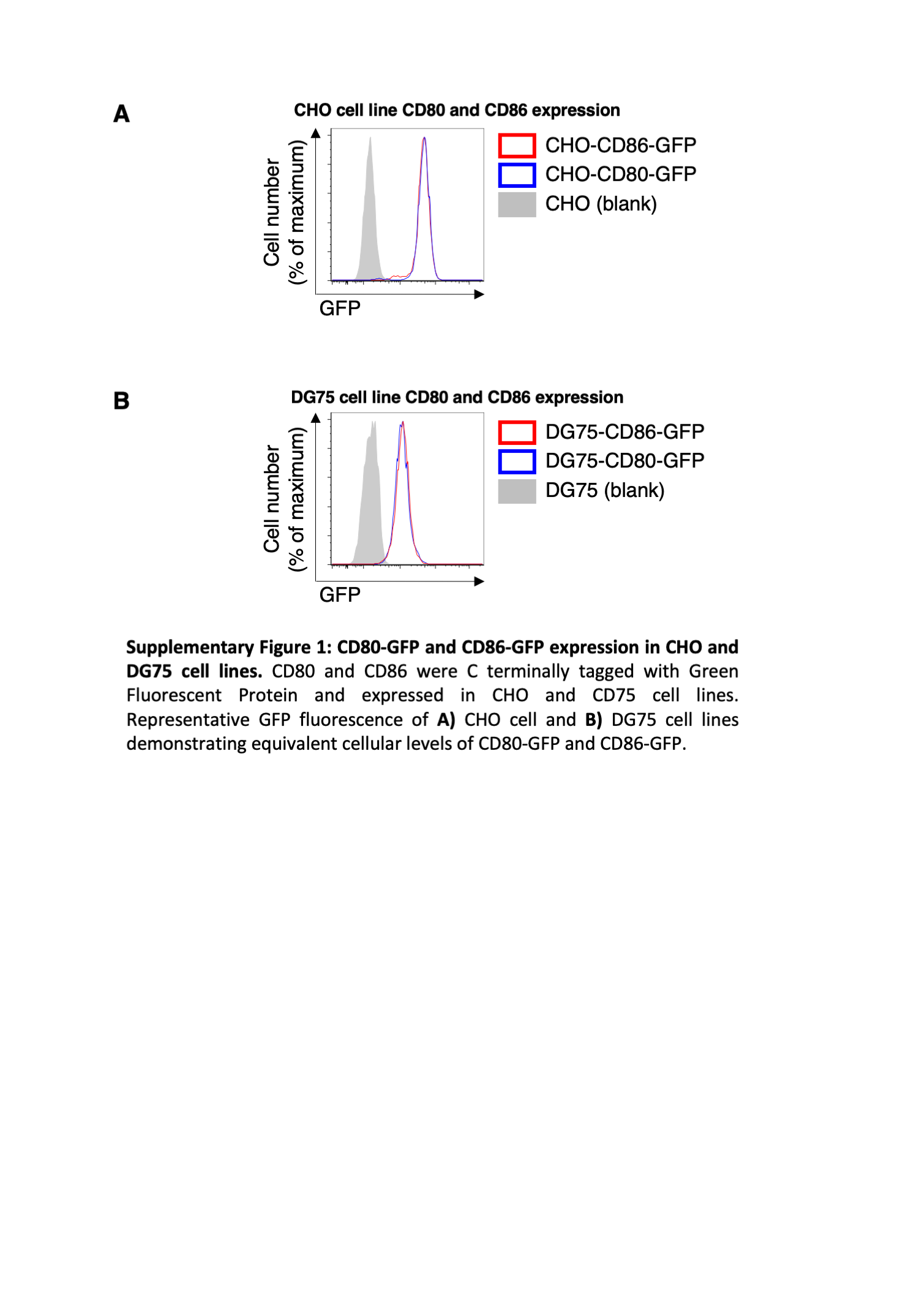

Supplement: Supplementary file 1 [file Image_1.tiff]

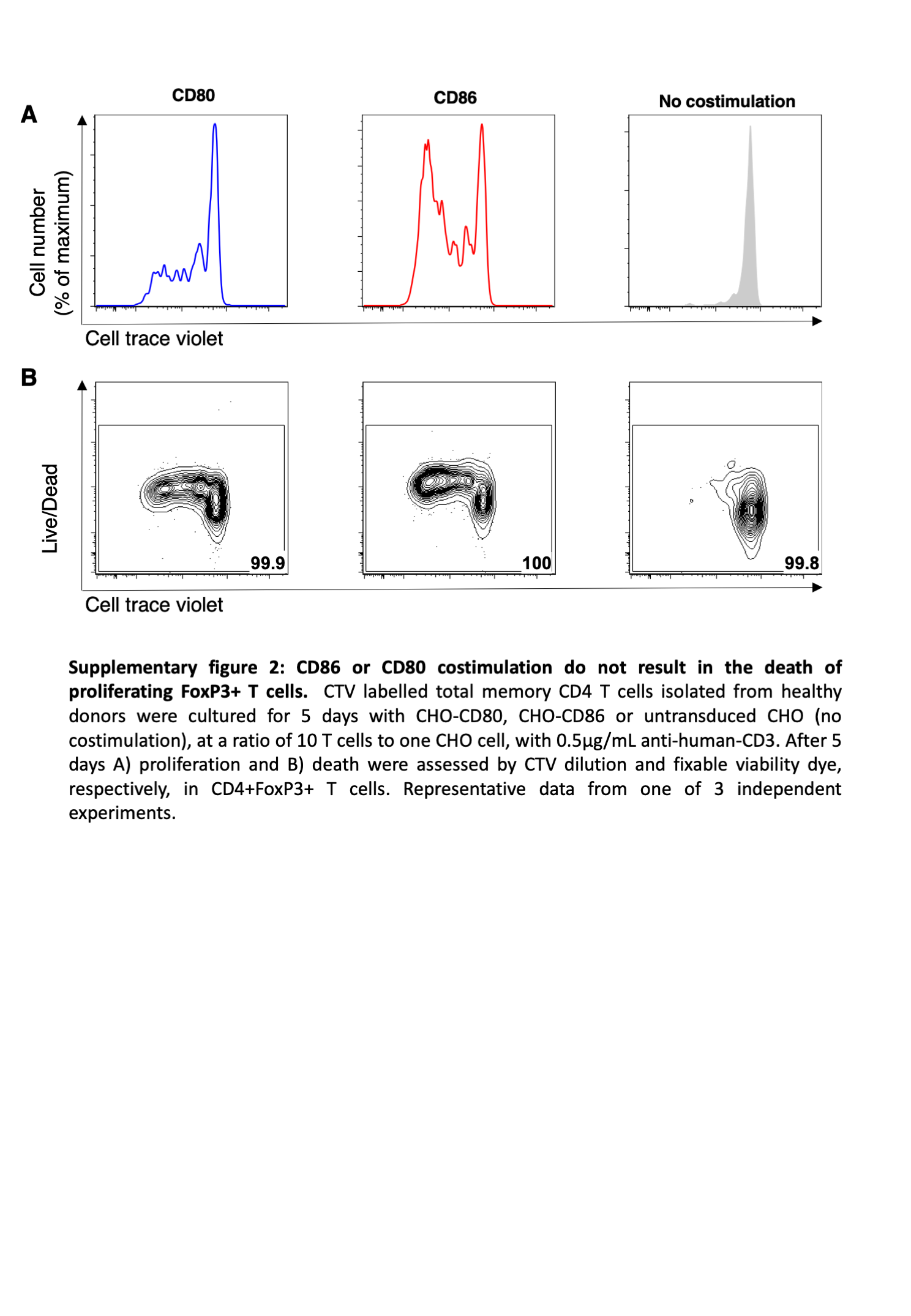

Supplement: Supplementary file 2 [file Image_2.tiff]

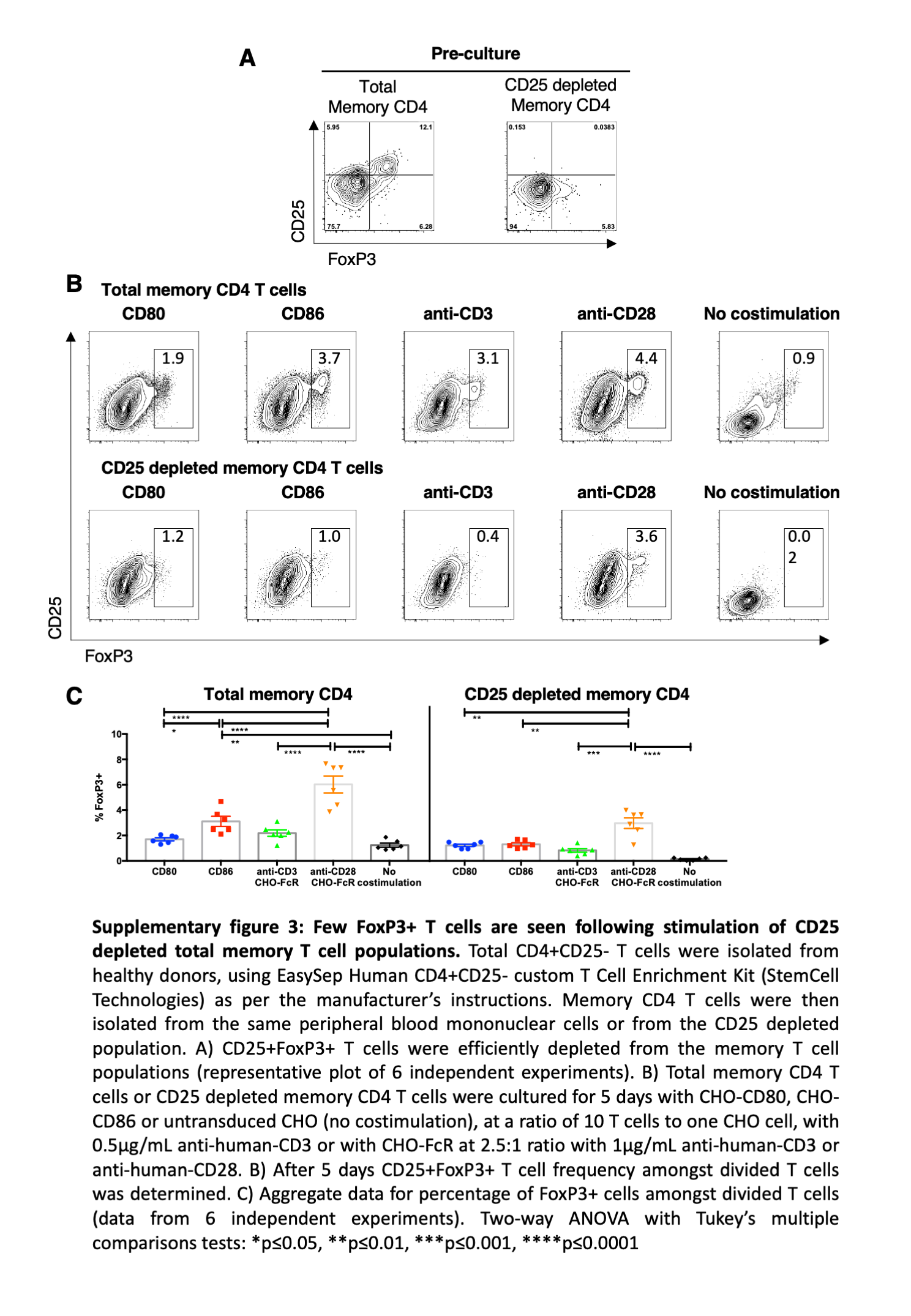

Supplement: Supplementary file 3 [file Image_3.tiff]

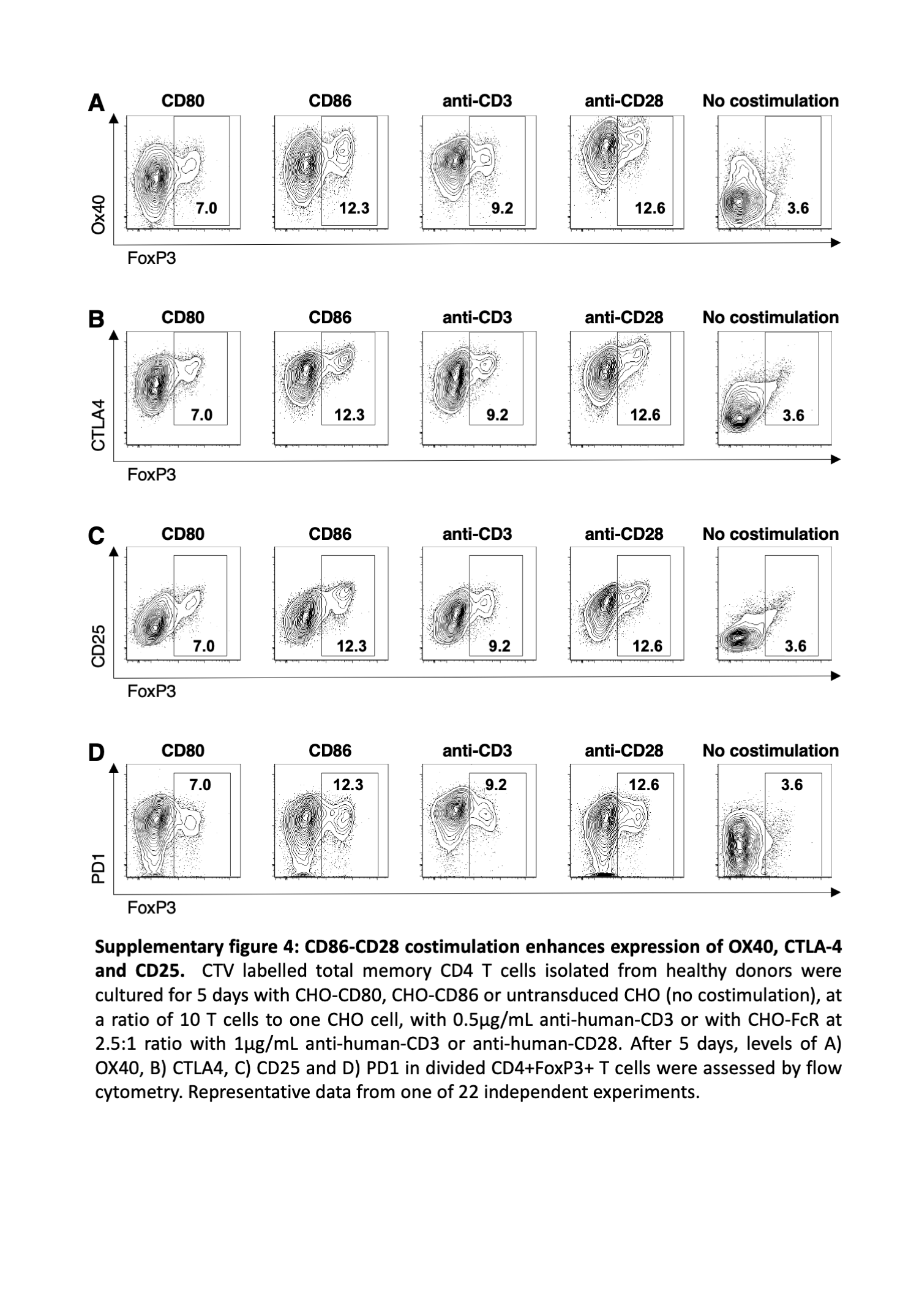

Supplement: Supplementary file 4 [file Image_4.tiff]
